# Retraction: Thyroid Ultrasound Findings in Children from Three Japanese Prefectures: Aomori, Yamanashi and Nagasaki

**DOI:** 10.1371/journal.pone.0308234

**Published:** 2024-07-29

**Authors:** The PLOS ONE Editors

Following publication of [1], concerns were raised that this article contains a substantial degree of overlap with a previously published article [2] with regard to the reported population sample, analyses performed, and overall conclusions. An editorial assessment by PLOS has established that the extent of overlap is such that this article [1] constitutes redundant publication.

The corresponding author stated that they had reused the data from [2] in [1] but that additional analyses were presented in [1]. However, the previous work [2] was not fully disclosed at submission, as required by *PLOS ONE*’s editorial policies [3, 4], and following review the *PLOS ONE* Editors and a member of the *PLOS ONE* Editorial Board assessed that adequate justification for the reuse of data was not provided.

The *PLOS ONE* Editors retract this article [1] because, per our editorial assessment, it does not meet *PLOS ONE*’s publication criteria (#2) [3], and because of the above concerns that remain unresolved and question this study’s compliance with the PLOS Ethical Publishing Practice policy [4].

SM and AO agreed with the retraction. NH, MI, HS, TN, SN, TA, MK, SS, SY, and NTakamura did not agree with the retraction. NO, YA, KK, and NTaniguchi, either did not respond directly or could not be reached.

## References

[1] Hayashida N, Imaizumi M, Shimura H, Okubo N, Asari Y, Nigawara T, et al. (2013) Thyroid Ultrasound Findings in Children from Three Japanese Prefectures: Aomori, Yamanashi and Nagasaki. PLoS ONE 8(12): e83220. 10.1371/journal.pone.0083220 24376666 PMC3871687

[2] Taniguchi N, Hayashida N, Shimura H, Okubo N, Asari Y, Nigawara T, Midorikawa S, Kotani K, Nakaji S, Imaizumi M, Ohtsuru A, Akamizu T, Kitaoka M, Suzuki S, Yamashita S, Takamura N; Investigation Committee for the Proportion of Thyroid Ultrasound Findings. Ultrasonographic thyroid nodular findings in Japanese children. J Med Ultrason (2001). 2013 Jul;40(3):219–24. doi: 10.1007/s10396-013-0456-1. Epub 2013 May 18. . 27277239

[3] https://journals.plos.org/plosone/s/criteria-for-publication#loc-2

[4] https://journals.plos.org/plosone/s/ethical-publishing-practice#loc-submission-and-publication-of-related-studies

